# Obituary

**Published:** 1905-03-15

**Authors:** 


### OBITUARY.

DR. W. L. ANDREWS, of Coldwater, Mich., died February 12th, at the age of 65 years. He was born in Fallsburg, N. Y. He studied dentistry for three years before the war, with Dr. Knapp, of Ft. Wayne, Ind. He enlisted in the Eleventh Indiana Heavy Artillary and served during the entire war. After his return from the army he practiced dentistry in Ft. Wayne and Kendallville, Indiana; in the latter place he found his wife, Miss Anna Thompson, whom he married in 1868. In 1873 he moved to Coldwater, Michigan, and practiced there until his death. He was a musician of more than average ability. He leaves a widow and one son. He was greatly respected for his social abilities in his home city.

J. R. CLAYTON, of Shelbyville, Ind., died January 13th, 1905. Dr. Clayton was well known as one of the prominent dentists in Indiana and Ohio. He was for several years professor of Prosthetic Dentistry in the Ohio College of Dental Surgery. He was also well known as a writer and scientific worker. We have no details of his death but get our information from the January *Digest*.

